# Implementing an Indeterminate Range for More Accurate Early Infant Diagnosis

**DOI:** 10.1097/QAI.0000000000002081

**Published:** 2019-05-29

**Authors:** Lara Vojnov, Martina Penazzato, Gayle Sherman, Anisa Ghadrshenas, Elaine J. Abrams, Meg Doherty

**Affiliations:** ^a^ HIV Treatment and Care, Department of HIV/AIDS, World Health Organization, Geneva, Switzerland; ^b^ Centre for HIV and STIs, National Institute for Communicable Diseases, Johannesburg, South Africa; ^c^ Department of Paediatrics and Child Health, Faculty of Health Sciences, University of the Witwatersrand, Johannesburg, South Africa; ^d^ ICAP at Columbia University Mailman School of Public Health, Columbia University, New York, NY; ^e^ Vagelos College of Physicians and Surgeons, Columbia University, New York, NY

***To the Editors:***

Since 2010, WHO has recommended that HIV virological testing be used to diagnose HIV infection in infants and children below the age of 18 months and that ART be started without delay upon the first positive result while a second specimen is collected to confirm the initial positive virological test result.^1^ Infants are, therefore, initiated on lifelong treatment after an initial positive result before the confirmatory test result is provided.^1^ Confirmatory testing is critical to verify patient identity (eg, exclude specimen switches or mislabeling) and exclude false-positive results (eg, due to specimen contamination or test specificity/positive predictive value being less than 100%).^2^ A primary challenge is that in some countries in sub-Saharan Africa, less than 10% of infants with initial positive test results receive a confirmatory test.

The potential for a false-positive result is of increasing concern as prevalence or transmission rates decrease.^3,4^ For example, in a setting with mother-to-child transmission rates of less than 5%, the positive predictive value of a highly sensitive nucleic acid-based technology decreased to nearly 70%.^5^ Fortunately, newer assays currently in use in the field have higher specificities with less dramatic decreases in positive predictive values.

Mother-to-child transmission rates have decreased considerably over recent years as option B+ and Treat All policies have been implemented,^6–8^ reaching as low as 2% in a high burden, treatment-experienced setting.^9–13^ This reduction in transmission can have a considerable impact on the positive predictive value of infant diagnostic assays, even those with nearly 100% sensitivity and specificity. It is important to note that this issue is primarily predicated on the prevalence/transmission rate in the tested population and not a technological shortcoming. Current assays on the market for infant diagnosis typically have specificities of greater than 98%;^14–17^ thus a more perfect assay is unlikely to significantly improve the positive predictive value in settings with low prevalence/transmission rates in the tested population.

Currently, most programs and laboratories interpret undetectable test results by the nucleic acid-based technology as negative and detectable test results as positive, relying on thresholds of detection provided by the manufacturers; however, there is increasing concern on how to interpret test results with low levels of viremia, particularly in the context of increasing exposure to antiretroviral treatment and prophylaxis. To date, there has been limited guidance on how to interpret low levels of viremia in test results. However, historically, one would hesitate to initiate lifelong treatment based on detection of the equivalent of very few viral copies per milliliter a true positive result. In this context, different approaches have been considered: guidelines in the United States suggest that infants should not be considered HIV-positive unless they have the equivalent of 5000 viral copies/mL or higher,^18^ whereas South Africa has introduced an indeterminate range that requires further testing before a definitive test result is provided and treatment is initiated.^19^

An indeterminate range is a range of viral copy equivalents that would be too low to be accurately diagnosed as HIV infection.^20^ Qualitative diagnostic assays do not always provide viral copies, but instead the polymerase chain reaction cycle threshold when amplification is observed. The cycle threshold is inversely correlated to the amount of virus in a sample.

To provide guidance on how to interpret diagnostic test results with low levels of viremia, a systematic review, meta-analysis, and cost-effectiveness model were developed.^21,22^ The systematic review of 32 studies using an indeterminate range^21^ found 14,753 non-negative test results, of which 2436 (16.5%, 95% CI: 15.9% to 17.1%) were indeterminate. Furthermore, one study that reported the final diagnoses of indeterminate cases found that 76% of infants with an initial indeterminate test result were negative upon retesting, suggesting that these infants were not HIV-positive despite the initial non-negative test result. These data indicate that in countries not implementing an indeterminate range to support result interpretation of infant diagnosis test results, 12.5% of non-negative results could be false positive on initial testing and those infants are potentially put on lifelong treatment unnecessarily.

Within the meta-analysis, a random effects model was run for primary data from 2017 and included 2077 data points from 5 studies across 4 countries (Botswana, Namibia, South Africa, and Uganda) using the Roche TaqMan v2 technology.^21^ Data provided included only patients with an initial non-negative test and a second test result from a new sample. To better understand the most accurate indeterminate range, true positivity and false positivity were calculated across various proposed indeterminate thresholds based on the initial test result (Table 1). For example, if an indeterminate threshold of ≥30 were used, meaning that all test results with a cycle threshold of 30 or greater would be classified as indeterminate, approximately 24% of true positives, but 98% of false positives would fall within that range. Based on available information at the time the guidelines were developed, the optimal indeterminate range is considered to be the equivalent of a cycle threshold of 33 on the Roche COBAS Ampliprep/COBAS TaqMan HIV-1 Qualitative Test v2.0 assay. This value represented the best trade-off between the proportion of infants living with HIV who would be incorrectly identified as indeterminate (approximately 8%) and the proportion of HIV-negative infants who would potentially start treatment unnecessarily (approximately 7%).

**TABLE 1.**
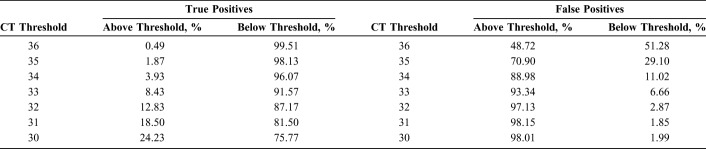
True Positivity and False Positivity Estimates 1 for Proposed Indeterminate Thresholds

The cost-effectiveness model compared the standard interpretation (no indeterminate range) with a variety of indeterminate range options and concluded that implementing an indeterminate range is far more effective than the standard of care across a variety of cycle threshold ranges.^22^ Furthermore, as the prevalence/positivity/transmission rate at each testing time point decreases, the cost-effectiveness of an indeterminate range increases and is more cost-saving than no indeterminate range.

A survey provided to program managers (n = 85), health care workers (n = 146), and people living with HIV (n = 587), established that over 85% of respondents in each group found implementation of an indeterminate range to be acceptable to prevent unnecessary lifelong treatment.^23^ Sixty percent or more respondents in each group preferred providing or receiving a full explanation of the meaning of an indeterminate result; however, several thought it would be critical to minimize confusion and concerns for caregivers. Implementing an indeterminate range would be equitable, as doing so would improve the quality of test results provided to caregivers and clinicians and, most importantly, prevent infants from being put on lifelong treatment unnecessarily.

Most countries already apply a national standard operating procedure when testing errors are encountered (such as device malfunction or insufficient or rejected specimen). Furthermore, a new study suggests that repeating an infant test on the same sample, if and when available, will resolve most (>95%) indeterminate test results.^24^ Therefore, before the health care facility is contacted to request that the mother and baby return to the facility for collection of a new sample, any indeterminate test should be repeat-tested on the same sample using additional available dried blood spots or remaining whole blood.^20,25^

Implementing an indeterminate range will support more accurate nucleic acid-based early infant diagnosis: It is likely that fewer infants would be put on lifelong treatment unnecessarily as most false positives would fall within the indeterminate range and receive additional testing before diagnosis rather than being identified as positive. However, some challenges remain to be resolved. First, the cycle threshold values provided by infant diagnostic assays on the market vary and cannot be directly applied between technologies or assays. Ideally, each assay manufacturer should implement the indeterminate range directly into their proprietary software. Test result readouts would then be positive, negative, or indeterminate. This would be particularly beneficial for point-of-care assays operated by lower cadres of staff to simplify result interpretation. For laboratory-based technologies, rapid determination of the equivalent cycle threshold or viremic values will allow for laboratories to implement the indeterminate range and subsequent repeat testing before software integration.

Furthermore, additional considerations may be necessary for countries using plasma as the sample type for infant testing rather than whole blood or dried blood spots, because the latter sample types typically capture and amplify intracellular nucleic acids that may increase the detected levels of virus. The age at which the child is tested and the timing of transmission may also affect the viral loads, because infants diagnosed at birth can have lower viral loads.^26^ However, no evidence informs and justifies differences in indeterminate range values based on the time of sample collection, and further research is needed. In addition, more research would be valuable on the feasibility of implementation and best messaging for health care workers and caregivers/mothers as well as optimal standard operating procedure for indeterminate test results.

Countries, particularly those with low transmission or infant diagnosis positivity rates, should strongly consider implementing an indeterminate range for better management of HIV-exposed infants. Furthermore, many of the issues presented here highlight the critical need for all infants with detectable virus to receive repeat testing on the same sample and for those who are clearly positive, confirmatory testing with a new sample. Finally, as mother-baby retention during the postnatal period is challenging and the proportion of transmission during the postnatal period is increasing,^8^ early infant diagnosis is growing increasingly complex. Continued testing throughout and after the exposure period is critical: Infant diagnosis should no longer be considered a one-time process.
